# Adherence Patterns and Dose Response of Physiotherapy for Rotator Cuff Pathology: Longitudinal Cohort Study

**DOI:** 10.2196/21374

**Published:** 2021-03-11

**Authors:** David Burns, Philip Boyer, Helen Razmjou, Robin Richards, Cari Whyne

**Affiliations:** 1 Sunnybrook Research Institute Sunnybrook Health Sciences Centre Toronto, ON Canada; 2 Division of Orthopaedic Surgery University of Toronto Toronto, ON Canada; 3 Working Condition Program Holland Orthopedic and Arthritic Centre Toronto, ON Canada; 4 Department of Physical Therapy University of Toronto Toronto, ON Canada; 5 Sunnybrook Health Sciences Centre Toronto, ON Canada

**Keywords:** rehabilitation, treatment adherence and compliance, wearable electronic devices, machine learning, rotator cuff

## Abstract

**Background:**

Physiotherapy is considered to be essential for the successful operative and nonoperative management of rotator cuff pathology; however, the extent to which patients adhere to assigned physiotherapy activities and how this impacts recovery is unknown.

**Objective:**

The purpose of this study was to measure the rate and patterns of participation in physiotherapy for rotator cuff disorders, assess the dose response between physiotherapy activity and recovery, and explore patient factors predictive of physiotherapy participation.

**Methods:**

We report a prospective longitudinal study of 42 patients undergoing physiotherapy for symptomatic rotator cuff pathology. The patients were issued a smartwatch that recorded inertial sensor data while they performed physiotherapy exercises both in the clinic and in the home setting. A machine learning approach was used to assess total physiotherapy participation from smartwatch inertial data. Primary outcomes were the Disabilities of the Arm Shoulder and Hand and numeric pain rating scale assessed every 4 weeks until 12 weeks follow-up. The relationships between participation, outcomes, and clinical patient variables were assessed in univariable analyses.

**Results:**

Mean physiotherapy exercise participation in clinic and at home were 11 minutes per week and 33 minutes per week, respectively, with patients participating in physiotherapy on 41% of days assigned to treatment. Home physiotherapy participation decreased significantly over time (*P*=.03). There was a statistically significant and clinically meaningful relationship between cumulative physiotherapy participation and recovery demonstrated by pain scores at 8 weeks (*P*=.02) and 12 weeks (*P*=.05) and disability scores at 8 weeks (*P*=.04) and 12 weeks (*P*=.04). Low patient expectations and self-efficacy were associated with low rates of physiotherapy participation.

**Conclusions:**

There was a low rate of participation in home shoulder physiotherapy exercise, and a statistically and clinically significant dose response of physiotherapy on treatment outcome in patients with rotator cuff pathology. The findings highlight the opportunity to develop novel methods and strategies to improve the participation in and efficacy of physiotherapy exercises for rotator cuff disorders.

**International Registered Report Identifier (IRRID):**

RR2-10.2196/17841

## Introduction

Rotator cuff pathology is a common cause of shoulder pain and disability [1,2] and is associated with significant utilization of health care resources [3] and societal economic costs [1]. Exercise-based physical therapy is an established first-line treatment for this condition [4-6] and is also an important element of rehabilitation following rotator cuff surgery [7,8]. Adherence to prescribed physical therapy exercise is considered to be essential for successful rehabilitation of both conservatively and operatively managed patients [9,10]. However, self-reported adherence to physical therapy is often poor (50%-70%) [9,11], particularly in the home setting [9,12,13] and in worker populations [10].

The concept of adherence, in the context of physiotherapy and rehabilitation, is multidimensional [14]. It includes behaviors such as attending clinical appointments, active participation in physiotherapist-supervised activities and home exercises, avoiding potentially harmful or contraindicated activities, and wearing protective or therapeutic devices. Adherence to the home component of physiotherapy exercise programs is important, as this activity calls for the greatest level of independent patient engagement in the rehabilitation process and typically represents most of the opportunity for physiotherapy exercise.

Objective measurement of adherence to home physiotherapy exercises remains an open problem [15]. Adherence diaries, in which patients self-report their independent exercises, are the recommended and most widely used measure of adherence to home exercise [15]. However, adherence diaries have significant limitations: The validity and reliability of the adherence diaries have not been established, they cannot measure or assess adherence to technique, and poor patient acceptability results in low rates of diary completion (60%-75%) [4,16,17].

The capacity to accurately and objectively measure home physiotherapy adherence would further our understanding of the rate and patterns of home physiotherapy adherence, the impact of adherence on recovery, patient motivations, and barriers to effective home physiotherapy engagement [12]. This understanding is a crucial first step to developing strategies to optimize home physiotherapy adherence.

Several technologies (chiefly wearable or video devices) have been developed and pilot tested for providing objective and complete assessments of adherence to home physiotherapy [13,18-26]. However, we are not aware of any that have been validated in a clinical population or used to obtain the necessary clinical insights. The common premise underlying a technical solution to adherence monitoring is using sensors to record patient home physiotherapy and having a computer algorithm classify activity type (and potentially evaluate technique).

Advances in the capabilities of wearable devices such as smartwatches and time-series machine learning methods present an opportunity to leverage robust and accessible technology for remote physiotherapy tracking. In our prior preclinical work [18], we demonstrated that shoulder physiotherapy exercise performed by healthy study participants can be accurately tracked using a smartwatch.

This paper presents the results of a study with the following objectives: (1) measure the rate and patterns of total (home and clinic) participation in rotator cuff physiotherapy, (2) assess the dose response between physiotherapy activity and recovery, and (3) explore patient factors predictive of physiotherapy participation.

## Methods

### Population

We performed a prospective longitudinal study of 42 patients with rotator cuff pathology. The inclusion criteria were (1) age ≥18 years, (2) diagnosis of unilateral rotator cuff tendinosis, shoulder impingement syndrome, or degenerative or traumatic rotator cuff tear, (3) planned conservative or operative management, (4) capacity to participate in home shoulder physiotherapy. The exclusion criteria were (1) upper extremity neurologic deficit, (2) bilateral symptomatic rotator cuff pathology, (3) failed surgical management of rotator cuff pathology.

The presence of rotator cuff pathology was determined clinically and confirmed with diagnostic imaging (magnetic resonance imaging or ultrasound).

### Registrations

Sunnybrook Health Sciences Centre institutional research ethics board approval was obtained for this study, and a protocol paper was published [27]. This manuscript represents a preliminary analysis of 42 patients out of 120 patients planned according to the protocol [27].

### Physiotherapy Treatment

Patients received 1-hour in-person shoulder physiotherapy sessions on a weekly basis and were assigned home exercises from a 19-exercise rotator cuff protocol by their treating physiotherapists (Multimedia Appendix 1). They were asked to complete their assigned exercises each day that they were not attending in-person physiotherapy. In addition to physiotherapy exercise, patients received other adjunct treatments at the discretion of their physiotherapist (heat, manual therapy, ultrasound, and electrotherapy). All physiotherapy services were funded either by the study or through worker’s compensation claims.

### Inertial Data Collection

Patients were provided with a Huawei 2 smartwatch (Huawei Technologies Co Ltd) to be worn on their affected extremity when performing prescribed shoulder physiotherapy exercise both at home and in the physiotherapy clinic. Inertial data (triaxial accelerometer, triaxial gyroscope, and triaxial magnetometer) data were recorded on the smartwatch at sampling rate of 50 Hz while being worn, then uploaded to a cloud storage server using a custom app. Inertial data were labeled during supervised physiotherapy for exercise type and number of repetitions.

### Primary Outcomes

A numeric pain rating scale (NPRS) [28,29] and the Disabilities of the Arm, Shoulder and Hand (DASH) score [30-32] were collected to measure the relationship between total (home and in-clinic) physiotherapy participation and patient recovery. These validated clinical outcome measures were assessed at baseline, 4 weeks, 8 weeks, and 12 weeks.

The NPRS pain scores were assessed using a 3-item survey with the following questions: (1) What is your pain at rest? (2) What is your pain with activity? (3) Over the past week, how bad has your pain been on average?

### Predictors of Adherence

To explore potential predictors of physiotherapy adherence [12,33-38], the following data were collected for each patient at recruitment: age, sex, BMI, baseline pain level (NPRS), baseline physical activity level (total hours per week of resistance and aerobic exercise), work status (working or not working), education, current income, ENRICHD Social Support Inventory score (perceived social support) [39], 2-item Pain Self-Efficacy Questionnaire (patient self-efficacy) [40], Patient Expectation Questionnaire score [41], and Hospital Anxiety and Depression Scale score [42]. This represents a subset of adherence predictors from those described in our protocol [27] with high response rate (>80%) and sufficient distribution among categorical variables.

### Machine Learning Algorithms

A supervised learning framework was used to train and validate a fully convolutional neural network (FCN) classifier [43,44] for detecting and differentiating physiotherapy exercise activity from the inertial data collected on the smartwatches. The raw data were preprocessed with an overlapping sliding window segmentation (10-second windows) to provide fixed-length input to the FCN classifier.

The FCN classifier was trained using labeled inertial data collected during supervised physiotherapy activity. The exercise-type data labels were mapped to simplified label consisting of the principal motion involved in that exercise. This mapping is detailed in Multimedia Appendix 1. The FCN model architecture is detailed in Multimedia Appendix 2.

Temporal data splitting was used to validate algorithm performance, using the last physiotherapy session for each patient in the test set, and all prior physiotherapy sessions for the training set. The training data set was augmented with data collected from 16 healthy volunteers as they performed routine activities of daily living including rest for 3 hours each. The test set was augmented with similar activities of daily living data from 4 healthy volunteers.

The FCN classifier performance was evaluated on the test set for (1) differentiating all physiotherapy activity from activities of daily living, and (2) differentiating between different physiotherapy activities.

### Physiotherapy Participation Tracking

Physiotherapy participation was assessed by processing a patient’s recorded inertial data using the trained FCN classifier for differentiating physiotherapy activity from activities of daily living. Patients, treating clinicians, and all research personnel were blinded to the physiotherapy participation rate measured by the system.

For the purposes of this study, daily physiotherapy participation was defined as the ratio of physiotherapy exercise measured for a patient to an expectation of 20 minutes per day (up to a daily maximum of 100%).

### Patient Experience

The patients were asked questions (Table 1) about their experience with smartwatch-based physiotherapy tracking.

**Table 1:** Survey questions.

Questions	Response options
How often did you use your smartwatch when you performed home physiotherapy?	Every time, most of the time, some of the time, rarely, never
What challenges did you have that prevented you from using the smartwatch when you performed your home physiotherapy?	None, battery, inconvenient, uncomfortable, other (specify):
How did having a smartwatch affect your participation in your home physiotherapy program?	I exercised a lot less; I exercised a little less; no effect; I exercised a little more; I exercised a lot more

### Data Analysis

#### Univariable Analyses

The relationship between outcomes (dependent variable) and cumulative participation was examined with least squares linear regression analysis after 4 weeks, 8 weeks, and 12 weeks of physiotherapy treatment.

The relationship between cumulative physiotherapy participation at 4 weeks (dependent variable) and individual baseline adherence predictor variables was explored with univariable statistical analyses. Parametric and nonparametric correlation analyses were conducted for continuous and ordinal predictor variables, respectively, and the 2-sample *t* test was used for binary predictors. Note, education was converted to a binary variable for analysis with a 2-sample *t* test.

#### Sample Size

This analysis reports on a cohort of 42 patients whose minimum treatment duration was 4 weeks, of whom, 42, 35, and 27 patients respectively received treatment for a duration of up to 4 weeks, 8 weeks, and 12 weeks, respectively.

We hypothesized the existence of moderate correlations (from 0.40 to 0.59) between improvement in outcomes and participation which requires a minimum of 19 to 45 patients to achieve a power of 0.8 to detect the relationship.

This interim analysis is not sufficiently powered to conduct robust multivariable analyses of adherence predictor variables.

## Results

### Patient Characteristics

**Figure 1:**
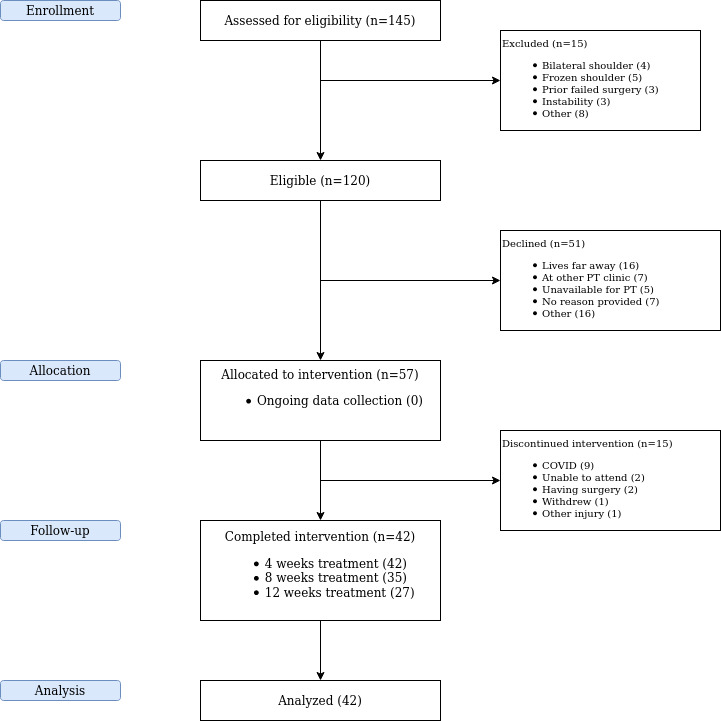
Patient flow through clinical study as of April 28, 2020. Physiotherapy treatment and data collection was suspended for 16 patients enrolled in the study due to physical-distancing measures imposed during the COVID-19 pandemic. PT: physiotherapy.

Patient flow through the study is shown in Figure 1, and a summary of patient characteristics is provided in Table 2.

**Table 2:** Patient characteristics.

Variable		Value (n=42)
Age (years), mean (SD)		45 (13)
**Gender, n (%)**		
	Male	15(36)
	Female	27 (64)
BMI (kg/m^2^), mean (SD)		26 (4)
Baseline physical activity (hours/week), mean (SD)		3.6 (4.4)
**Currently working status, n (%)**		
	Currently working	19 (45)
	Not currently working	23 (55)
**Active worker’s compensation claim, n (%)**		
	Yes	9 (21)
	No	33 (79)
**Rotator cuff tear, n (%)**		
	Full thickness	13 (31)
	Partial thickness	12 (29)
	No tear	17 (40)
**Smoking, n (%)**		
	Currently smokes	1 (2)
	Previously smoked	6 (14)
	Never smoked	35 (83)
Income, median (CAD)		40,000-60,000
**Education, n (%)**		
	Professional or university degree	21 (50)
	College or no degree	21 (50)
**Diagnostic imaging, n (%)**		
	Magnetic resonance imaging	23 (55)
	Ultrasound	26 (62)
	None	2 (5)
**Physiotherapy treatment adjuncts, n (%)**		
	Manual therapy	23 (55)
	Heat therapy	18 (43)
	Ultrasound	17 (40)
	Electrotherapy	3 (7)
Perceived social support (ENRICHD Social Support Inventory), mean (SD)		26 (5)
Pain self-efficacy (Pain Self-Efficacy Questionnaire), mean (SD)		8.0 (3.2)
Patient Expectation Questionnaire, mean (SD)		18 (4)
Anxiety (Hospital Anxiety and Depression Scale), mean (SD)		7.0 (5.5)
Depression (Hospital Anxiety and Depression Scale), mean (SD)		5.5 (3.5)

### Inertial Data Collection

Inertial sensor data were collected using a Huawei 2 smartwatch from each study participant during in-clinic supervised physiotherapy and in the home setting. In total, 1275 hours of inertial data were collected. Of this, 290 hours were collected during supervised physiotherapy. Technical issues impacting inertial data collection occurred with an incidence of 4% (101/2376 attempted recordings). The majority of these errors occurred due to Wi-Fi connectivity problems with the hospital network.

### Primary Outcomes

#### Pain

**Figure 2:**
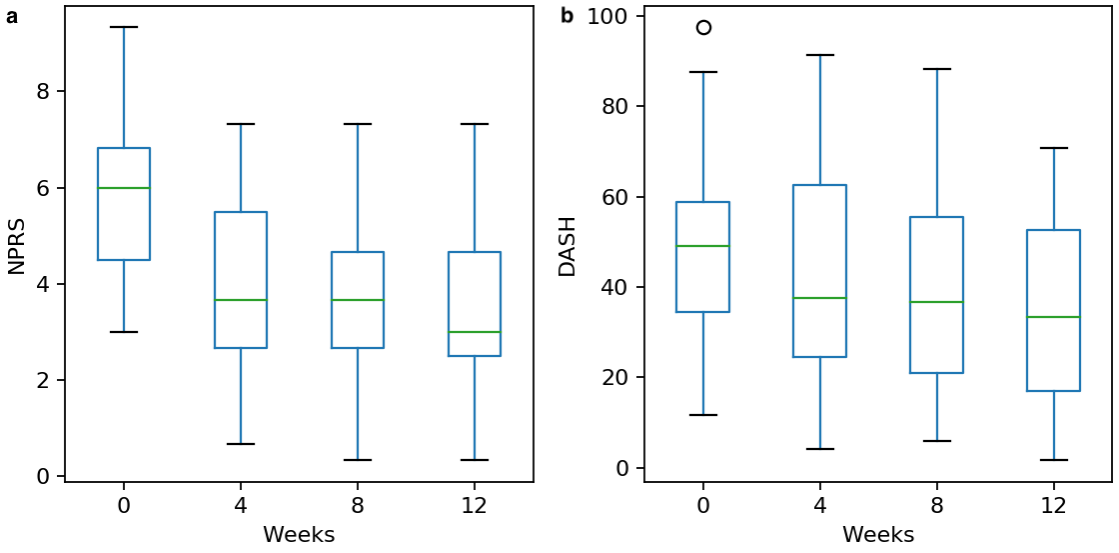
Clinical improvement in (a) pain and (b) disability. NPRS: numeric pain rating scale. DASH: Disability of the Arm, Shoulder, and Hand.

The pain outcome was modeled as the mean of the 3 NPRS survey items. There was a reduction in NPRS scores from a mean of 5.2 (SD 1.9) at baseline to a mean of 3.4 (SD 1.7) at 12 weeks (*t*=6.8, *P*<.001), with 93% of patients (39/42) experiencing at least some improvement. Improvement in pain score exceeded the minimal clinically important difference (MCID) for the NPRS (1-2.2) [45-47] in 48% to 78% of patients (Figure 2).

#### Disability

There was a reduction in DASH score from a mean of 44 (SD 21) at baseline to a mean of 35 (SD 20) at 12 weeks (*t*=6.8, *P*<.001), with 81% of patients (34/42) experiencing at least some improvement. Improvement in DASH scores exceeded the MCID (10.83 [48]) in 48% of patients (20/42) (Figure 2).

### Machine Learning Validation

**Figure 3:**
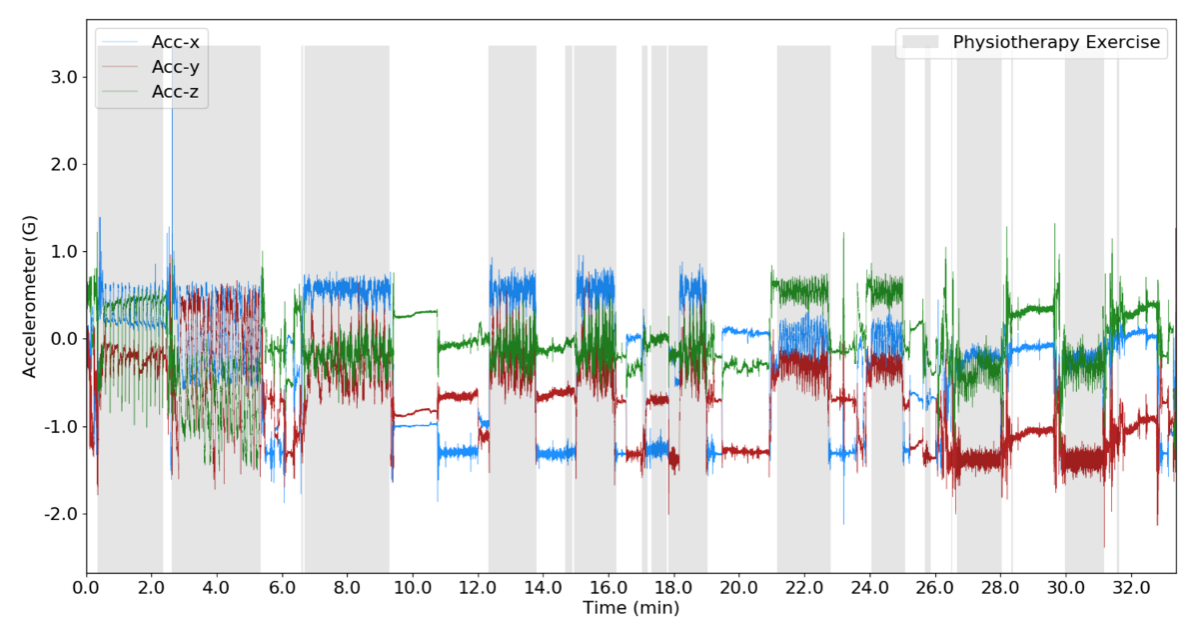
Predicted binary classification of physiotherapy exercise activity and interexercise rest periods overlaid on triaxial accelerometer data. The repetitive oscillatory patterns of exercise are correctly identified by the model.

For the binary classification task of differentiating physiotherapy activities from rest and activities of daily living, the FCN model achieved high levels of performance (accuracy 0.95; sensitivity 0.94; specificity 0.97; area under the receiver operating characteristic curve 0.99). An example demonstrating the physiotherapy classifier correctly predicting exercise and rest intervals during a physiotherapy session is shown in Figure 3.

For the multiclass problem of differentiating individual physiotherapy exercises types, the FCN classifier achieved an accuracy of 0.90 and an F1 score of 0.82.

### Physiotherapy Participation

**Figure 4:**
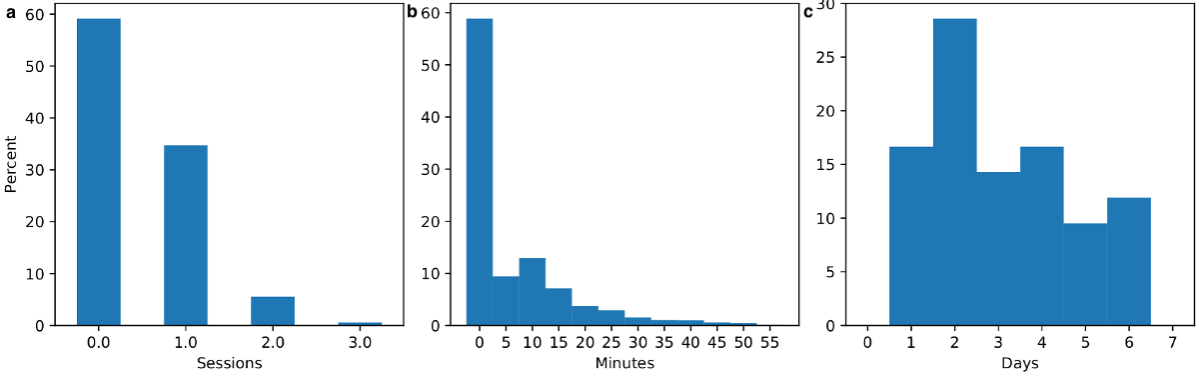
Total physiotherapy participation by (a) sessions per day, (b) minutes per day, and (c) days per week.

Patients participated in physiotherapy on 41% of the days on which they were assigned treatment (1388/3386 patient-days), usually for a single physiotherapy session with exercises lasting between 5 to 15 minutes. Figure 4 depicts the distribution of total physiotherapy participation rates in terms of sessions per day, minutes per day, and days per week.

**Figure 5:**
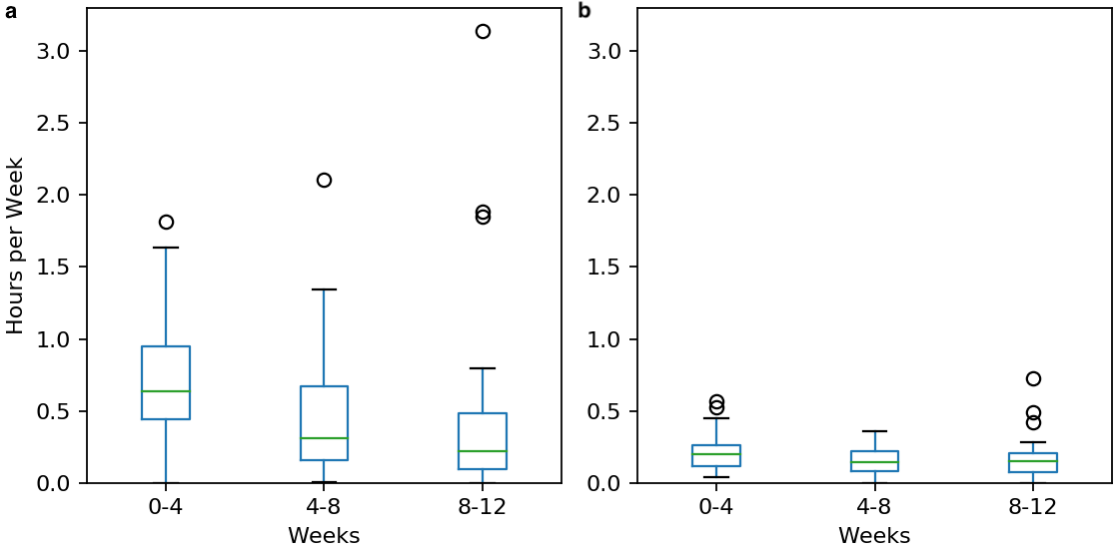
Changes in physiotherapy participation for (a) home and (b) clinic settings.

Home physiotherapy participation decreased over time (see Figure 5), from a median 38 minutes per week in the first 4 weeks of treatment to a median of 13 minutes per week in weeks 8 to 12 (*t*=2.3, *P*=.03). There was no statistically significant decrease in physiotherapy participation in clinic, which remained at approximately 10 minutes throughout the 12 weeks of therapy (*t*=1.7, *P*=.09).

**Figure 6:**
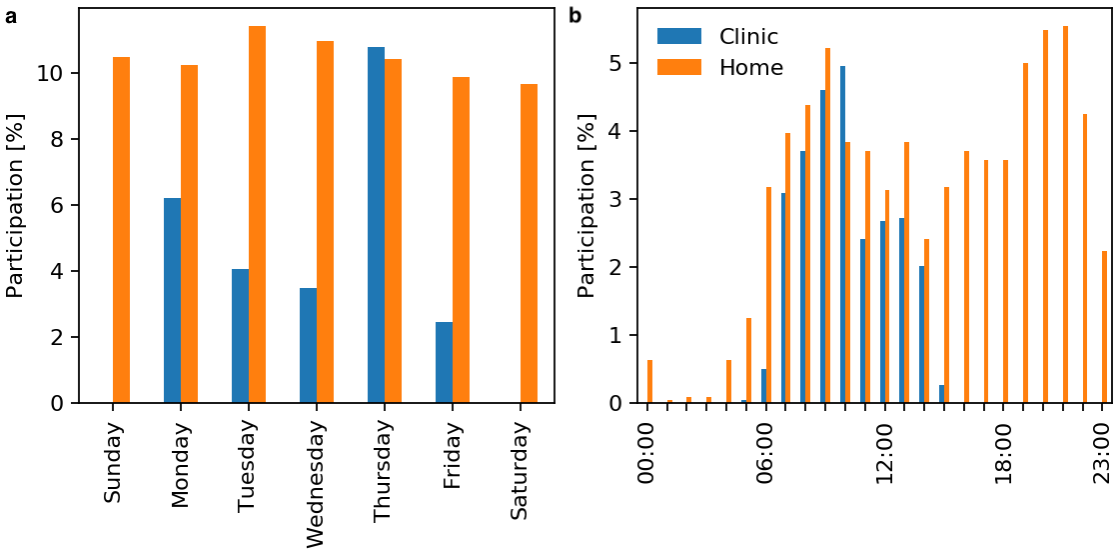
Patterns of physiotherapy participation for (a) days of the week and (b) time of the day (from midnight to midnight the next day).

Daily patterns of physiotherapy participation are shown in Figure 6. Home physiotherapy participation is spread equally across days of the week. There was a bimodal distribution of home physiotherapy participation, peaking in the morning (10 AM) and evening (9 PM). Differences in patterns of home physiotherapy participation based on sex, work status, and age are provided in Multimedia Appendix 3 (Figures S1-S3).

### Participation and Recovery

**Figure 7:**
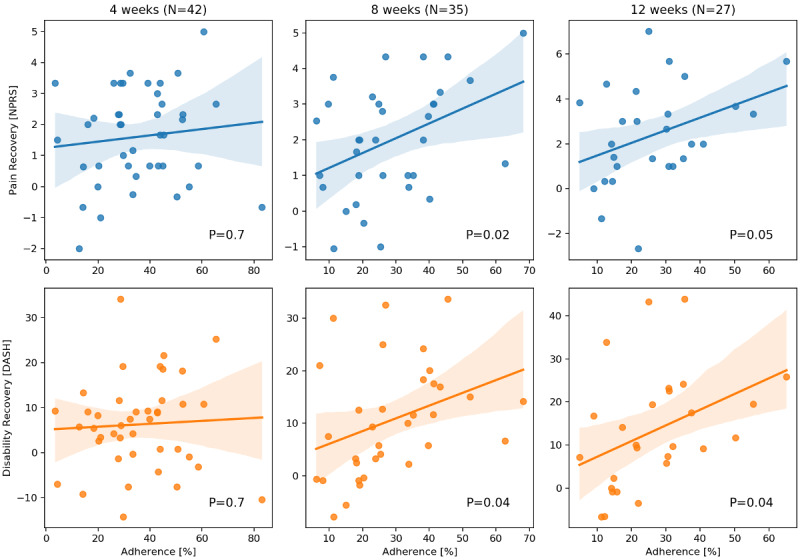
Physiotherapy dose response. Participation was defined as the ratio of physiotherapy exercise measured for a patient to an expectation of 20 minutes per day (100%).

The relationship between total physiotherapy participation and recovery in pain and disability scores is shown in Figure 7.

There was a relationship between participation and improvement in DASH score at 8 weeks (*R*=0.35, *P*=.04) and 12 weeks (*R*=0.39, *P*=.04) but not at 4 weeks (*R*=0.06, *P*=.70). The magnitude of this effect at 12 weeks (slope 0.37) was such that improvement in participation by 29% or more was correlated with clinically important differences in recovery (MCID 10.83 [48]).

There was a relationship between participation and improvement in pain score at 8 weeks (*R*=0.40, *P*=.02) and 12 weeks (*R*=0.37, *P*=.05) but not at 4 weeks (*R*=0.11, *P*=.48). The magnitude of this effect at 12 weeks from the regression slope (0.056), was such that improvement in participation of 18% to 39% or more was correlated with clinically important differences in recovery (MCID 1-2.2 [45-47]).

### Predictors of Adherence

Descriptive statistics and univariable analyses for the potential adherence predictors collected for exploratory analysis are detailed in Table 3. The following predictors were found to be positively correlated with physiotherapy participation: patient expectations for recovery (*P*=.007), self-efficacy (*P*=.04), lower anxiety scores (*P*=.03), and greater income (*P*=.03).There was also a nonsignificant trend for greater physiotherapy participation in older patients (*P*=.06).

**Table 3:** Univariable analysis of patient variables with cumulative physiotherapy participation over 4 weeks of treatment.

Patient variables				Value		*P* value
**Continuous, Pearson correlation**						
	Age (years)			0.33		0.06
	BMI (kg/m^2^)			0.01		0.95
	Baseline pain (numeric pain rating scale)			–0.01		0.94
	Baseline physical activity (hours/week)			0.21		0.21
**Ordinal, Spearman correlation**						
	Social support (ENRICHD Social Support Inventory)			0.19		0.24
	Pain self-efficacy (Pain Self-Efficacy Questionnaire)			0.32		0.04
	Patient Expectation Questionnaire			0.42		0.007
	Anxiety (Hospital Anxiety and Depression Scale)			–0.34		0.03
	Depression (Hospital Anxiety and Depression Scale)			–0.21		0.19
	Income			0.35		0.03
**Categorical, adherence mean (SD)**						
	**Sex**					0.41
		Male	39 (12)			
		Female	34 (19)			
	**Work status**					0.62
		Working	37 (19)			
		Not working	35 (13)			
	**Worker’s compensation**					0.51
		Active claim	32 (18)			
		No claim	36 (16)			
	**Education**					0.44
		Professional or university degree	38 (17)			
		College or no degree	34 (17)			

### Patient Experience With Physiotherapy Tracking

There were 26 respondents to the patient experience survey. Patients reported using the smartwatch during home physiotherapy every time (11/26), most of the time (12/26), or some of the time (3/26). Challenges encountered with the technology were related to battery life (8/26), remembering to use the smartwatch (1/26), and the recording function (1/26). Most patients reported exercising at home as result of wearing the smartwatch in this study either a lot more (5/26) or a little more (14/26). The other respondents reported that smartwatch use did not affect their home physiotherapy participation (7/26).

## Discussion

Our study’s findings echo previous findings in the literature based on patient self-report, indicating that there is high rate of poor participation in home physiotherapy [9,11]. We also found that there was a significant decline in physiotherapy participation over the course of treatment. The low level of participation that we observed was particularly notable given that many patients (19/26, 73%) indicated they were participating more than they would otherwise without tracking, despite blinding of both patients and health care providers to the tracking results.

The most important finding of this analysis is the dose response observed for cumulative physiotherapy participation at 8 and 12 weeks of treatment. It is generally assumed that if a treatment program is efficacious, adherence to treatment yields improved results. There are existing data to support this notion in the context of physiotherapy. Holmgren et al [49] demonstrated that a specific exercise protocol supervised by physiotherapists was superior to self-directed range of motion exercises performed at home. Østerås et al [50] demonstrated a dose response to rotator cuff rehabilitation, with high-dose (greater frequency and intensity) exercise training producing greater benefits than low-dose training under the direct supervision of a physiotherapist. To our knowledge, our study is the first to directly and objectively measure the dose response to shoulder physiotherapy exercises performed by patients independently at home. We found that there was a correlation between relatively modest increases in home physiotherapy participation and clinically meaningful improvements in pain and disability outcomes.

The common paradigm for physiotherapy treatment delivery is the same as that of this study. Patients are typically trained in the required exercises by their treating physiotherapist and periodically reassessed; however, they are responsible for performing the majority of their exercise-based therapy independently. The physical-distancing measures imposed by the current COVID-19 pandemic have even further restricted patient access to supervised in-person exercise physiotherapy. The major limitation of the current approach to treatment delivery is highlighted by the mounting evidence that the independent exercise required of patients often does not occur and that many patients are thus not receiving the full benefit of this important and effective treatment. Finding a feasible solution to this issue remains an open problem.

To improve independent physiotherapy exercise participation in the home setting first requires an understanding of patient motivations and barriers to adherence. There is a growing body of literature that has carefully considered these issues, using patient self-reported home exercise adherence or clinic attendance as the principal instruments for data collection [12,51-55]. The 2010 systematic review by Jack et al [12] reported low baseline levels of physical activity, low adherence to exercise under supervision, low self-efficacy, depression, anxiety, helplessness, poor social support, greater perceived number of barriers to exercise, and increased pain during exercise as factors related to physiotherapy adherence.

Our study found that patients with greater expectations for recovery and greater self-efficacy had better participation in physiotherapy. While our patients, on average, had reasonable expectations for recovery with their physiotherapy treatment (survey score out of 23: mean 18, SD 4), patients who were not confident in the benefit of the assigned program were less likely to participate in it independently. This insight could motivate better assessment and communication of treatment expectations in our program. The conceptual importance of patient expectations in relation to placebo and nocebo effects is also worth considering, as patients with higher expectancies are likely to have higher treatment outcome scores independent of other factors [56].

Various strategies for improving self-efficacy [57] that may be worthwhile to explore in the context of exercise-based physiotherapy also exist. We also found higher physiotherapy participation in patients with lower anxiety scores and higher personal income. While these 2 factors are not necessarily easily modifiable, this insight may assist clinicians in identifying patients at risk of poor adherence.

There was also a trend (statistically not significant) of greater physiotherapy participation in patients who were older. We found no relationship between physiotherapy participation with sex, BMI, baseline physical activity, baseline pain, perceived social supports, depression, work status, worker’s compensation status, or education level. With a sample size of 42, this study does not rule out these variables as potentially important predictors of physiotherapy participation. However, our data suggest that a moderate or weak effect size would be expected for these predictors if they are indeed found to be statistically significant in a larger population sample.

Further work is required to better understand patient motivations, barriers to adherence, and the efficacy of different methods for improving engagement in order to develop a coherent strategy for tackling this problem. We feel that objective and quantitative measurement of participation is important in all these arenas, both as a research tool and as part of a suite of derived strategies to motivate and drive further engagement. The relationship between modifiable predictors and physiotherapy participation shown in this exploratory analysis suggests that interventional strategies designed to target these areas (expectation and self-efficacy) may be promising avenues to pursue to increase participation and recovery.

Our deep learning approach was successfully validated for accurately tracking shoulder physiotherapy participation using inertial data collected on a smartwatch. The smartwatch proved to be an accessible method for data capture, with patients reporting smartwatch use during all or most home physiotherapy sessions and minimal challenges. An advantage of using wearable devices for activity tracking is that they are unobtrusive and easy to use anywhere, unlike some solutions based on video capture. A limitation of wearable devices is that they are only suitable for tracking physiotherapy exercises involving the limb or anatomic region on which the device is worn. This interim analysis has focused on total physiotherapy participation which represents one element in the broader notion of treatment adherence. We intend to also consider assessment of effort and adherence to specific exercise techniques, however, this future work depends on capturing inertial data from a larger sample of patients.

This study has a number of limitations. Our sample size of 42 patients limited our ability to detect weak relationships in the data or perform a meaningful multivariable analysis. The sample size was further reduced in the analysis of the 8- and 12-week data, due to the suspension of the study as COVID-19 pandemic protocols took effect. The COVID-19 pandemic interrupted our ability to provide ongoing in-clinic physiotherapy treatments to a number of our study participants, who therefore received shorter duration treatment than they otherwise would have. However, we felt it important to share the data that we have gathered thus far given its relevance to current COVID-19 physical-distancing restrictions, which impose a greater need for patients to engage in independent physiotherapy exercise. In addition, due to the small sample size, responses to multiple questions in the patient expectations survey that addressed different concepts were summed and analyzed as one single variable. This is based on an assumption of approximation to an interval scale (for all questions), which allows the latent variable of the overall expectation to be represented with a single summed value.

There are limitations with respect to the smartwatch and machine learning approach that we used for digital measurement of physiotherapy participation. The accuracy of our digital measurement depended on the correct use of the technology by patients. Patients were asked to wear their smartwatch during every physiotherapy session and to not wear it otherwise. Instances in which patients either neglected to wear their smartwatch or charge its batteries, as well as errors in the recording app introduced discrepancies between the digital participation measure and actual participation that could bias results. However, the impact of these effects is likely modest since 88% of patients (37/42) indicated that they used their smartwatch during all or most physiotherapy sessions, and we encountered few errors with the technology.

Instances in which patients wore their smartwatch outside of performing physiotherapy activities is another potential source of measurement error. Our FCN machine learning model was validated to accurately discriminate physiotherapy activity from activities of daily living, including resting, working at a computer, walking, jogging, etc. A limitation of our approach is that we could not validate the model to discriminate physiotherapy activities from all possible activities and did not specifically assess model performance against other fitness activities (eg, swimming, yoga, weight training) that might have similar inertial signals to physiotherapy. The discriminative performance of the FCN model would likely be degraded on activities outside of the training set, which could impact results for patients who chose, against instruction, to wear their smartwatch during such activities.

A further limitation the study design is that correlations were found between home physiotherapy participation and recovery support but do not prove a causal relationship. Any patient baseline variables related to both outcome and adherence, as well as uncontrolled treatment differences could bias the results. A multivariate analysis would be required to determine if physiotherapy participation is an independent correlate of clinical outcome, which would lend support to the causal notion. Unfortunately, our small sample size precluded such analysis. Ultimately, a prospective interventional study design would be the best approach to evaluate this question.

A final limitation of our study, and one that could impact future interventional study designs, is that tracking in itself could be considered an intervention with a measurable impact on adherence and recovery. A 3-arm randomized controlled trial with an untracked control, a passive (noninterventional) tracked control, and a tracking-enabled engagement platform would be the most rigorous path forward to study an adherence intervention.

In-home shoulder physiotherapy exercise participation was poor, and this was correlated with inferior pain and disability treatment outcomes for patients with rotator cuff pathology. While participation is correlated with higher expectations for recovery, better self-efficacy, lower anxiety, and higher income, further work is required to better understand the reasons for poor participation and develop methods to optimize home physiotherapy adherence.

## Appendix

Multimedia Appendix 1 Exercise motion mapping.

Multimedia Appendix 2 Machine learning model.

Multimedia Appendix 3 Patterns of home physiotherapy participation.

## Abbreviations

COVID-19: coronavirus disease 2019
DASH: Disabilities of the Arm, Shoulder and Hand
FCN: fully convolutional neural network
MCID: minimal clinically important difference
NPRS: numeric pain rating scale
