# Supplementary material for: Adherence Patterns and Dose Response of Physiotherapy for Rotator Cuff Pathology: Longitudinal Cohort Study
Source: JMIR Rehabil Assist Technol. 2021 Mar 11;8(1):e21374. doi: 10.2196/21374 (PMC8082948; doi:10.2196/21374)
Supplement: Multimedia Appendix 1 [file rehab_v8i1e21374_app1.docx]

**EXERCISE MOTION MAPPING**

The list of physiotherapy exercises used in our rotator cuff protocol is detailed in Table 3. Exercises are grouped by “motion type”, which was used as a target variable to improve the performance of the machine learning model used to differentiate physiotherapy activity from activities of daily living. The mapping between exercise and motion type is also detailed in Table 3.

| **Exercise** | **motion type** |
| --- | --- |
| 1. Assisted shoulder flexion (lying) | flexion |
| 2. Assisted shoulder flexion (standing) | flexion |
| 3. Assisted shoulder external rotation (sitting) | ER |
| 4. Assisted shoulder internal rotation (standing) | IR |
| 5. Active shoulder flexion (standing) | flexion |
| 6. Press up against wall (standing) | press-up |
| 7. shoulder girdle stabilization with elevation (standing) | flexion |
| 8. Resisted lat pull down (standing) | pull-down |
| 9. Resisted row (standing) | row |
| 10. Resisted external rotation (standing, adducted) | ER |
| 11. Resisted internal rotation (sitting, adducted) | IR |
| 12. Active shoulder abduction (standing) | abduction |
| 13. Assisted shoulder internal rotation (side-lying) | IR |
| 14. Resisted lat pull down (standing, external-rotation) | pull-down |
| 15. Resisted seratus anterior (sitting) | press-up |
| 16. Resisted shoulder scaption (sitting) | flexion |
| 17. Resisted triceps pull down (standing) | elbow-extension |
| 18. Resisted external rotation (standing, abducted) | ER |
| 19. Pushup | press-up |

**Table 3:** Physiotherapy exercises used in our rotator cuff protocol, with mapping to motion type target for training the FCN physiotherapy classifier.
