# Supplementary material for: Adherence Patterns and Dose Response of Physiotherapy for Rotator Cuff Pathology: Longitudinal Cohort Study
Source: JMIR Rehabil Assist Technol. 2021 Mar 11;8(1):e21374. doi: 10.2196/21374 (PMC8082948; doi:10.2196/21374)
Supplement: Multimedia Appendix 2 [file rehab_v8i1e21374_app2.docx]

**MACHINE LEARNING MODEL**

The FCN machine learning model architecture used in this study is an established strong baseline for time series classification [43,44]. The FCN model core (Figure 11) consists of 1D convolutional layers, with rectified linear unit (ReLU) activation, and batch normalization (BN). Regularization is achieved using dropout applied at each layer. Global average pooling is used after the last convolutional layer to reduce the model sensitivity to translations along the temporal axis. Our open source implementation is available at: <https://github.com/dmbee/fcn-core>.


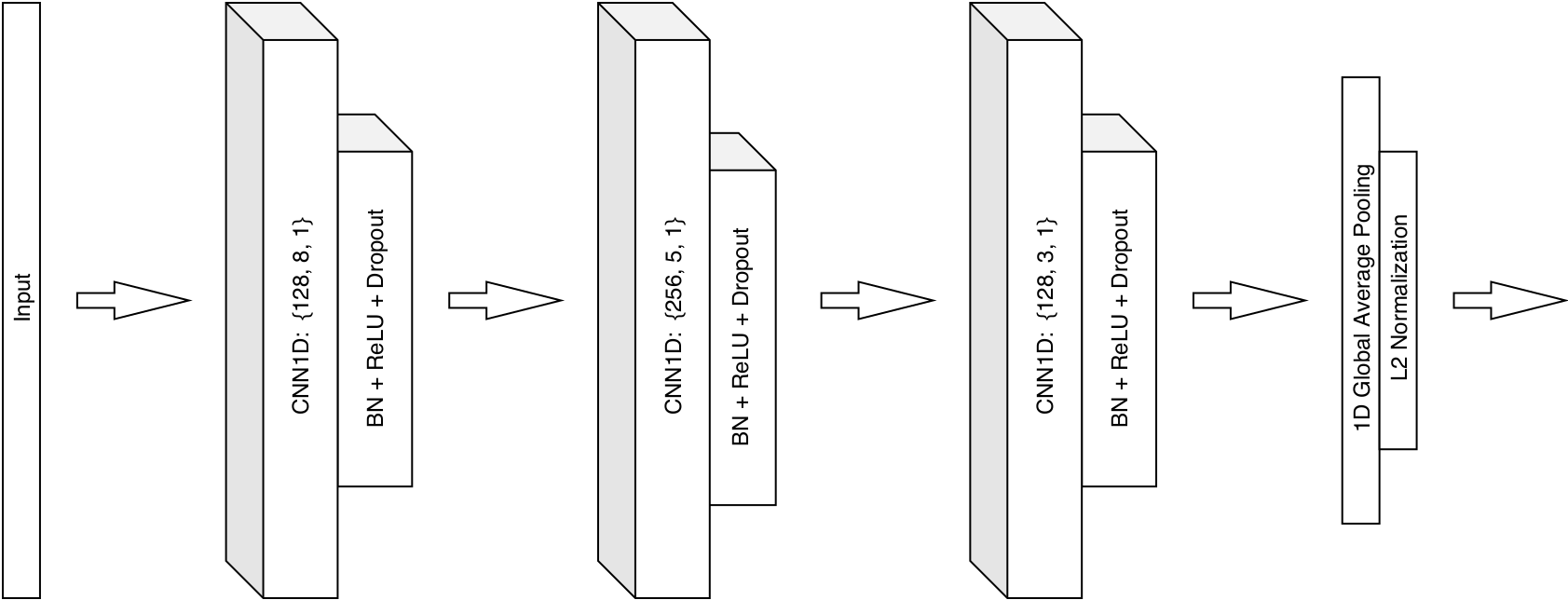


**Figure 11:** Fully convolutional network (FCN) model core. 1D convolutional layers are defined by (filters, kernel size, stride). A dropout ratio of 0.3 was used at each layer.
