# Supplementary material for: Adherence Patterns and Dose Response of Physiotherapy for Rotator Cuff Pathology: Longitudinal Cohort Study
Source: JMIR Rehabil Assist Technol. 2021 Mar 11;8(1):e21374. doi: 10.2196/21374 (PMC8082948; doi:10.2196/21374)
Supplement: Multimedia Appendix 3 [file rehab_v8i1e21374_app3.docx]

**PATTERNS OF HOME PHYSIOTHERAPY PARTICIPATION**

Figures 8, 9 and 10 depict how patterns of home physiotherapy participation varied based on sex, work status, and age.


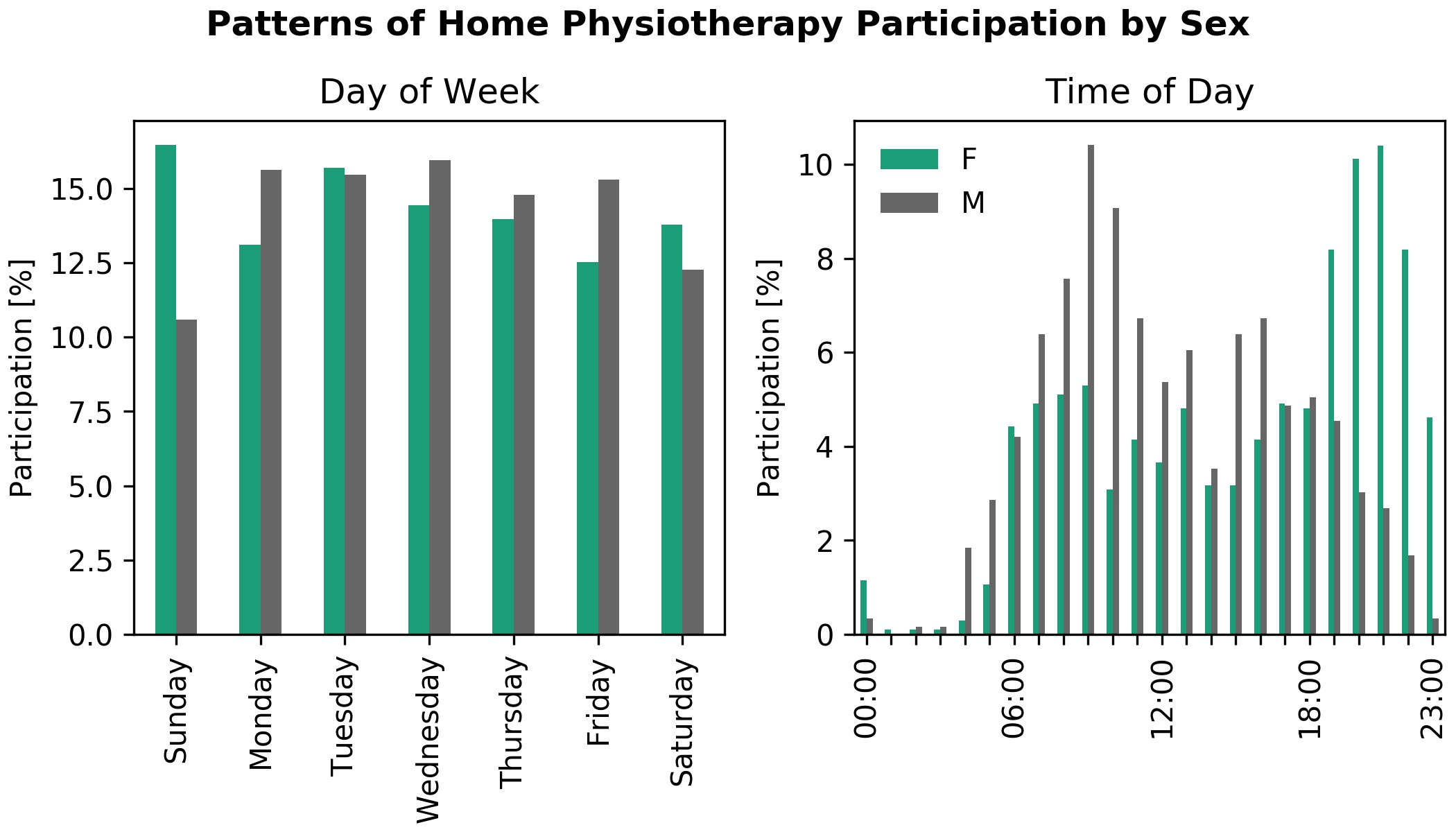
**Figure 8:** Patterns of home physiotherapy participation stratified by sex. Women tended to exercise later in the evening than men.


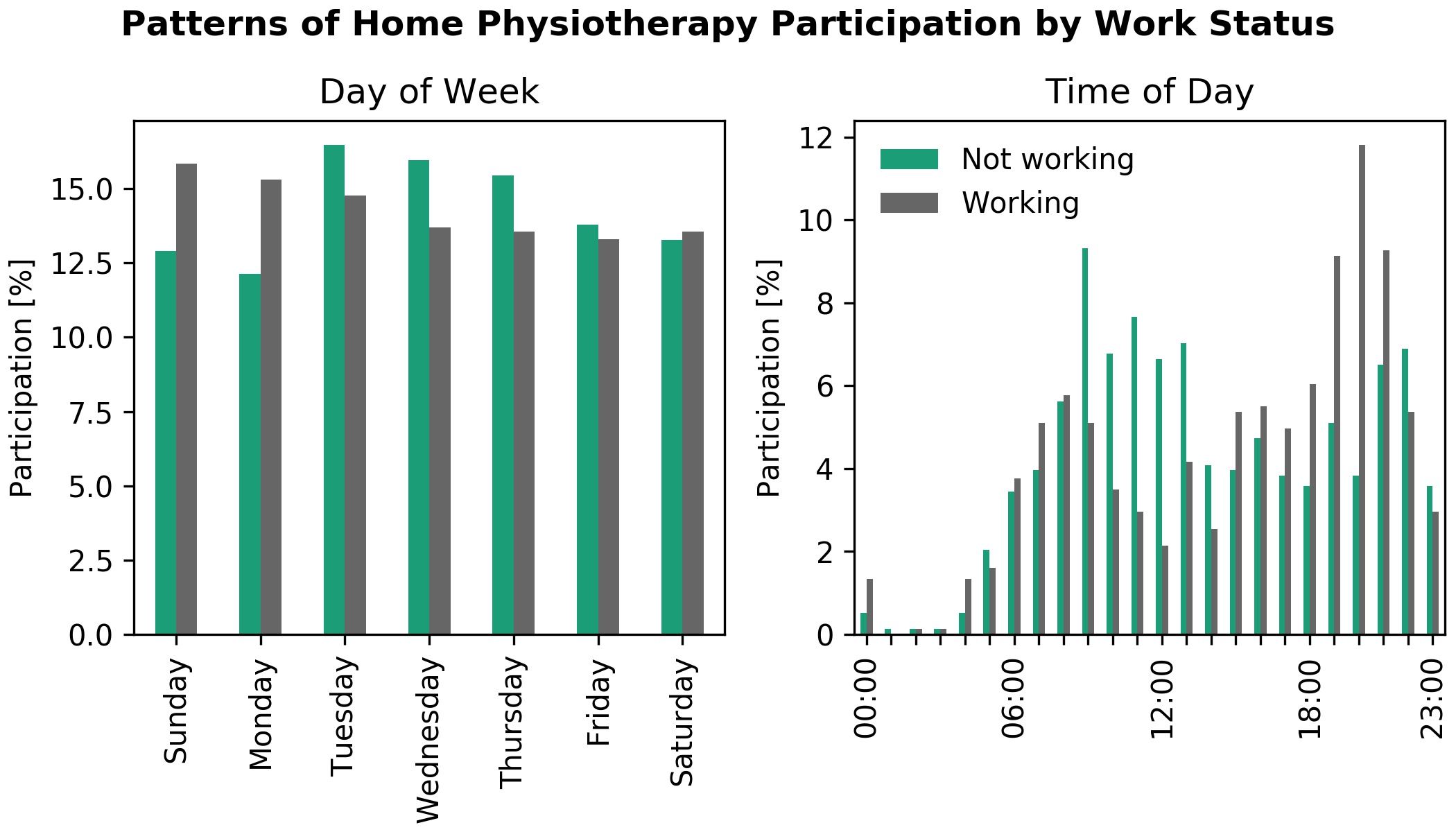
**Figure 9:** Patterns of home physiotherapy participation stratified by work status. Workers tended to exercise later in the evening than non-workers.


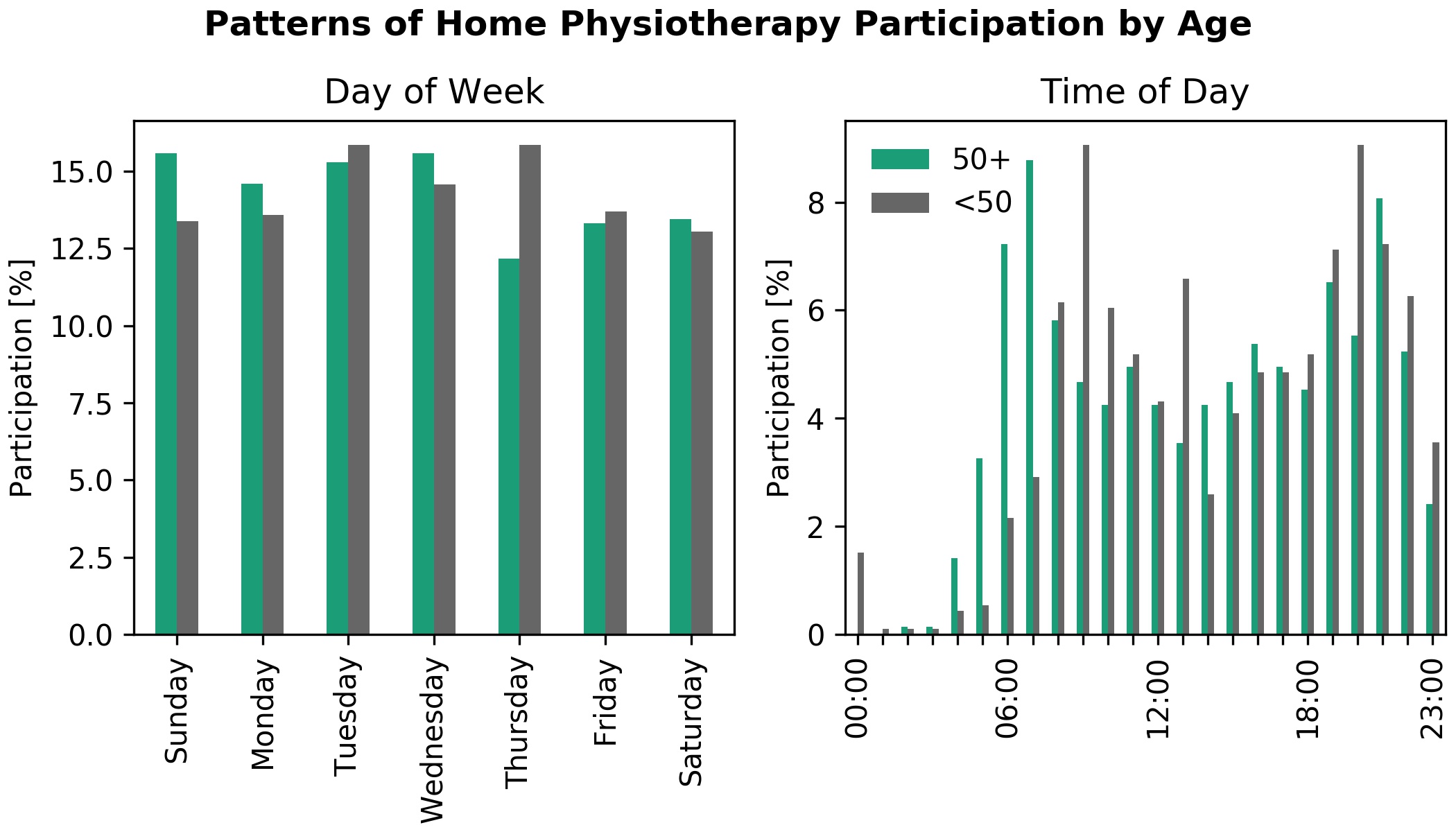
**Figure 10:** Patterns of home physiotherapy participation stratified by age. Older patients tended to exercise earlier in the morning.
